# Bibliometric Analysis: The Effects of Triclosan on Human Health

**DOI:** 10.3390/toxics10090523

**Published:** 2022-09-01

**Authors:** Rachel K. Papavasilopoulos, Sanghoon Kang

**Affiliations:** Department of Biological Sciences, Eastern Illinois University, Charleston, IL 61920, USA

**Keywords:** triclosan, bibliometrics, human health

## Abstract

Triclosan (TCS) is a widely used chemical whose effects on human health remains elusive. TCS may play a role in a variety of health issues, including endocrine dysfunction, irregular embryonic development, and immune suppression. It is possible that TCS’s penetrative abilities across all body barriers, including the blood–brain barrier, may make bioaccumulation the primary driver of these issues. In addition, chronic overuse of this chemical in everyday life may further contribute to the already increasing problem of antibiotic resistance. TCS research has steadily increased since its transition from medical to commercial use over the last 50 years. However, there are some clear gaps in the depth of this research as the safety of this agent is not fully agreed upon. The Food and Drug Administration recently issued regulatory rules regarding TCS in some commercial products; however, it is still found in a variety of goods marketed as “antimicrobial” or “antibacterial”. The purpose of this bibliometric study is to analyze research trends in this field and determine the amount of global attention TCS has received as to its relevancy in human health. Documenting and determining research concentration trends related to this field may outline where additional research is most necessary, as well as demonstrate the most valuable research produced and its relation to the advancement of our understanding of TCS. We found there to be a shift in research from TCS and its role in medical environments, to research based on the indirect effects of TCS through environmental contaminations, such as the propagation of antibiotic resistance. This shift was coupled with an increase in global research related to this field and identified China as a significant contributor. Although TCS has received notice, the simple fact of its continued use in so many common products, as well as the unclear understanding of its direct health impacts, reinforces the need for additional and more conclusive research before it has possible irreversible effects on our environment and health.

## 1. Introduction

Triclosan (TCS), or 5-chloro-2-(2,4-dichlorophenoxy) phenol, is an antimicrobial agent, registered pesticide, and known toxicant, which is a frequent and common ingredient in household products, such as deodorant, liquid soaps, cosmetics, and furniture [1]. TCS was originally developed for use in hospital settings, in products such as surgical sutures, scrubs, implants, and medical devices, and since then has been introduced into an array of products marketed as “antimicrobial” or “antibacterial” for commercial use [2] In September of 2016, the Food and Drug Administration (FDA) issued a regulation on the use of Triclosan in certain products with inconclusive evidence on its long-term effects on human health per the advice of the Nonprescription Drugs Advisory Committee (NDAC) of US FDA [3]. This decision by the FDA was based on public comments and all data that came to the attention of the FDA regarding this issue, in addition to the advice from the NDAC. TCS, along with 18 additional active ingredients were labeled as not “generally recognized as safe and effective” (GRASE), since their clinical benefits were not significant enough to outweigh their potential toxic and carcinogenic effects [3]. Products such as soaps, cosmetics, and shampoos fall under the jurisdiction of FDA regulations, but this ruling only applies to antiseptic washes for non-medical settings, such as “antibacterial” hand soaps for at-home use. Thus, TCS is still widely used in dozens of commercial products and goes unregulated, especially in items that do not fall under the FDA’s regulatory controls, such as furniture, clothing, and kitchenware, including knives and cutting boards.

TCS targets the phospholipid membrane through its detergent-like property that affects the stability of lipid structures [4]. Some bacteria species have developed mechanisms to resist the toxic activity of TCS, such as non-specific multidrug resistance (MDR) efflux pumps. At high concentrations of TCS, MDR efflux pumps are activated, essentially nullifying any effects of the agent in bacterial destruction or colony growth inhibition [5]. TCS may exert antibiotic resistance by upregulating MDR efflux pumps at high concentrations of TCS exposure [5]. However, the relationship between long-term, low concentrations of TCS exposure and efflux pumps remains unknown in bacterial populations [6].

Research on the effectiveness of TCS remains inconclusive. TCS has been reported to be especially useful in the prevention of the spread of disease in healthcare settings, effectively performing as an antimicrobial agent. Most notably, the use of TCS-coated sutures proves especially efficient in the prevention of surgical site infections post closure, when used in decontaminated surgical environments [7]. However, for use outside of medical settings, such as in soap, TCS has not been proven to be more effective than traditional soap [8]. Antibacterial soap (0.3% TCS) proved to have high bactericidal efficacy when bacteria were under continuous exposure of 24 h, any time below that failed to prove statistically significant efficacy. This is especially important when considering the CDC recommendations for hand-washing techniques, which consider 20 s of thorough hand washing under warm water sufficient [9]. It should be noted that traditional soap does not pose any of the AR-related issues that compounds such as TCS do while showing compatible efficacy in removing microbes from hands.

Antibiotic and antimicrobial overuse seeps into every form of our daily life and continually puts us at higher risk of bacteria that we do not have the ability to fight when resistance emerges [10]. Although the in vivo implications of TCS in humans are not as widely studied as the effects in animal populations, available studies of both have outlined the detrimental effects it could have on human health [11]. The least understood aspect of TCS is bioaccumulation and how that may relate to long-term health effects. Multiple urinalysis studies indicate the presence of TCS in subjects from various locations around the world. In the U.S., 75% of the 2517 participants were found to have tested positive at concentrations of 2.4–3790 μg/L for TCS in their urine, much higher than the baseline detection level of 2.3 μg/L [12]. Analogous studies in China show average levels of TCS in urine to be 100 μg/L in a random sample representative of the average population [13]. TCS retention has also been analyzed in oral mucosa, skin, and placental structures. A 64.5 mM alcoholic solution containing TCS was applied to rat skin, and after 24 h of constant application, 23% of the solution penetrated the skin surface after analysis [14]. This solution resembled the common cosmetic products we encounter every day. In human models, an average of about one-third of that concentration is absorbed into the skin surface [14]. Similarly, only 5.9% ± 2.1% of a 2% TCS cream dose remained in the urine after dermal application [15]. In oral mucosa, 0.660 mg of a 4.50 mg dose of TCS in a mouth rinse (0.03% concentration) was absorbed and retained after use [16]. Another study found that TCS remained on toothbrush bristles for an average of an additional two weeks raising participants’ exposure by 7–12.5 times the expected amount [17]. Although the dermal absorption of TCS is relatively low (around 3–7%), its 21-h half-life increases the likelihood of bioaccumulation due to a risk of prolonged exposure from a large combination of different products via different administration routes, further proved by the consistently high detection of TCS in various urinalysis studies [2].

TCS was also detected in neonatal cord blood. A study in New York City showed that 100% of urine samples from 181 expectant mothers and 51% of cord blood samples from their infants contained TCS [18]. TCS’s ability to cross the placental barrier leads to the question of its effect on neonatal development and subsequent behavior problems in children exposed to TCS early on in life. TCS exposure has been linked to decreased embryonic formation and implantation rates in patients with the highest levels of TCS urine concentration rates in comparison with other participants, as well as increased rates of spontaneous abortion, likely due to the inhibition of estrogen sulfotransferase activity resulting in placental thrombosis [19,20]. Perinatal exposure to TCS in mouse models may be linked to the disruption of neurogenesis and neuronal growth, which is then further correlated with behavioral and social problems after birth, including decreased memory, and increased anxiety-related behaviors [21]. Phthalates, including TCS, may increase behavior related to autism in animal models, and may be considered a risk factor for autism spectrum disorders [22]. Decreased hippocampal function and decreased spatial memory efficiency have been reported due to TCS’s inhibition of long-term potentiation and modification of neuronal plasticity in hippocampal neurons [23].

In the 2016 ruling on the safety of TCS, the FDA determined that although TCS has been shown to cause significant endocrine disruption in rat models, because of the physiological differences between humans and rats, TCS should pose no risk to human health [3]. They then follow this statement with the comment that there is a significant lack of human-related studies and therefore an accurate conclusion on the dangers of TCS to human endocrine health cannot be made. In rat models, TCS exposure was shown to have harmful effects on thyroid function by accelerating cell mutation and cell death [24]. In addition, decreased levels of circulating thyroxine and triiodothyronine hormones and the overall suppression of thyroid activity due to TCS have been reported as well [25,26]. Studies related to the direct effects of TCS on human endocrine health remain sparse and conflicted [20]. Compared to endocrine dysfunction, TCS’s effect on immune function may be slightly better understood. The activation of NLRP3 inflammasome in rat tissues due to exposure to TCS may result in increased allergies [27]. This has been mirrored in studies comparing the use of household products containing TCS and increased incidences of allergic responses [28]. The direct mechanism of how TCS affects the immune system of animal models is not fully understood, although some theories have recently developed. Increased TLR4 activation due to TCS exposure in rats has been shown to lead to inflammation and tumorigenesis in the colon [29]. Additionally, TCS may impair effector cell and cytokine expression; increase IL-1β, TNF-α, and TSLP expression; and increase B-cells, dendritic cells, and T-cell circulation [30]. An 87% suppression of natural killer cells’ function, following the 24-h application of TCS suggests the agent has an impact on immune system suppression [31]. Overall, due to the physiological differences between humans and rodents, more studies on human subjects would be desired for a more direct implication of TCS. TCS may also impact the gut microbiome by decreasing bacterial diversity, such as that seen within the digestive systems of 14-week-old male mice [32]. This decreased gut bacterial growth from TCS may play a larger role in an organism’s immune system, as decreased microbiome richness may contribute to pro-inflammatory responses [32].

Altogether, TCS is a potential risk to human health in development, neuropsychology, immunity, the emergence of AR, and others; however, it remains prevalent throughout our everyday use of common commercial products. The status and trends of research on the effect of TCS on human health have not been systematically analyzed in recent years. Therefore, the purpose of this bibliometric study is to analyze research trends in this field and determine the amount of global attention it has received regarding its effects, thus identifying a potential gap in global research trends for the suggestion of future research endeavors.

## 2. Materials and Methods

### 2.1. Data Collection and Processing

**Figure 1:**
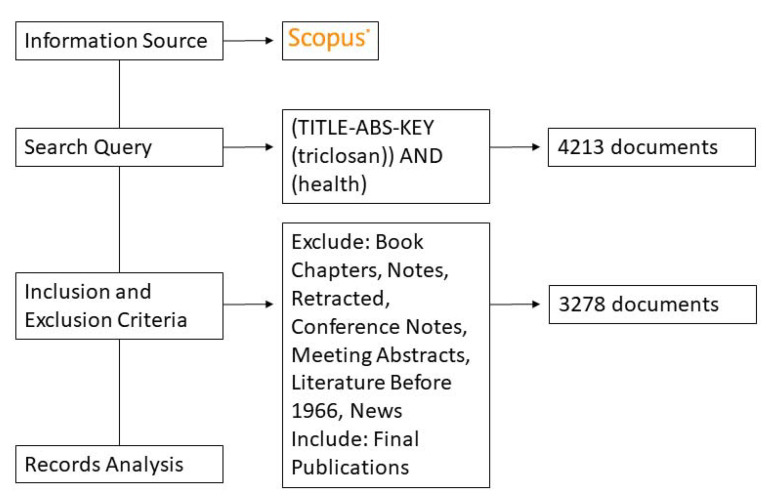
Data Collection and Processing.

The workflow demonstrates the data collection process used in this analysis (Figure 1). All data were retrieved from the public database, Scopus^®^, which broadly encompasses research from multiple scientific fields. A search of the data was performed on 8 November 2021. All data were imported into Microsoft Excel and VOSviewer for analysis [33]. The search query included all publications with the words “Triclosan” and “Health” in either the “titles, abstracts, or keywords” in the entirety of the database. This search included all titles, abstracts, and keywords similar to the search as well, such as “TCS” or other abbreviations. An initial broad search yielded a total of 4213 documents related to the search query. Further excluded from this data was any research categorized as book chapters, notes, retracted work, conference notes, meeting abstracts, or news items. Only included were final and completed peer-reviewed publications. The final yield was 3278 usable documents for analysis. The documents were downloaded in Excel binary file format (.xls) for further analysis. Research collected included all literature spanning from 1 January 1966 to 11 August 2021. The entire dataset is available in the Supplementary Materials (Table S1).

### 2.2. Bibliometric Analysis Methods

This study involves the analysis of research related to the TCS and its effect on human health in a broad range of concentrations. TCS dates back to its original patent in 1966 filed by the company Ciba-Geigy and was then introduced into medical settings in 1973 [1]. For this analysis, research into TCS produced only after 1966 was included. Basic analysis was performed directly in Microsoft^®^ Excel. This includes growth trends, citations per year, main researchers, and most prominent journals that published TCS research. Further visual analysis was then carried out in VOSviewer 1.6.17. VOSviewer is a software tool for constructing visuals related to bibliographic information [33]. This includes data about citation and co-citation analysis, co-authorship analysis, and global network analysis.

## 3. Results and Discussion

### 3.1. Research Growth and Characteristics

**Figure 2:**
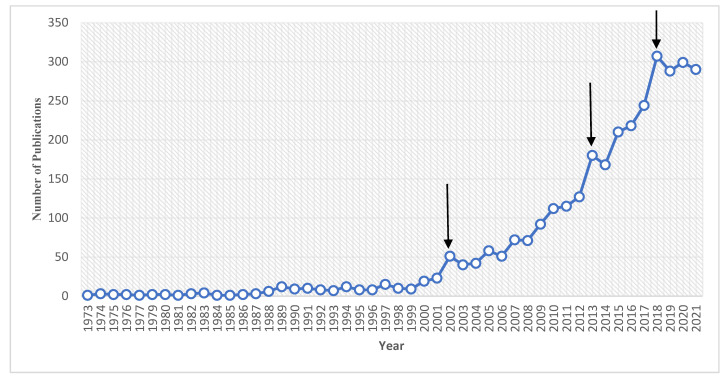
TCS-health-related publications per year. Arrows indicate spikes in the number of publications produced in correlation with significant events in this field (2002, 2013, and 2018).

A total of 3278 documents that fit the search criteria were analyzed. Research growth was analyzed between the dates of 1 January 1973, and 11 February 2021. Although TCS was patented in 1966 for use in medical procedures and environments, research related to TCS on human health did not emerge until 1973 [1]. Publications between 1973 and 2021 yielded a total of 3219 documents. Research in this field has steadily increased over 50 years, with significant spikes in 2002, 2013, and 2018 (Figure 2). From 1973 until 2001, TCS-health-related research was relatively low, with a total of 184 publications. TCS use in its early production years was primarily for medical practice usages, such as surgical procedures for the use of disinfection of the skin, surgical sutures, and implanted medical devices [2]. Between the years 2002 and 2012, 831 articles were published, an almost 5-fold increase in just 10 years from the prior 30 years. TCS was introduced into household products, mainly hand soaps, in 1987 [34]. In addition, in 1997, the FDA approved the low dose use of TCS in Colgate Total toothpaste for the prevention of dental health issues, such as gingivitis [35,36]. This transition from medical use into more prevalent commercial use, may at least partly account for the notable increase in research on its effects on human health. Rates of publications going forward remained at a similarly stable increase with one additional spike beginning in 2018, followed by a plateau. This plateau followed the FDA’s ban on TCS in liquid soaps for public consumption, as well as the monitoring of TCS levels in other products [3]. The period spanning 2013–2021 saw a total of 2204 published articles related to TCS.

### 3.2. Subject Analysis

**Figure 3:**
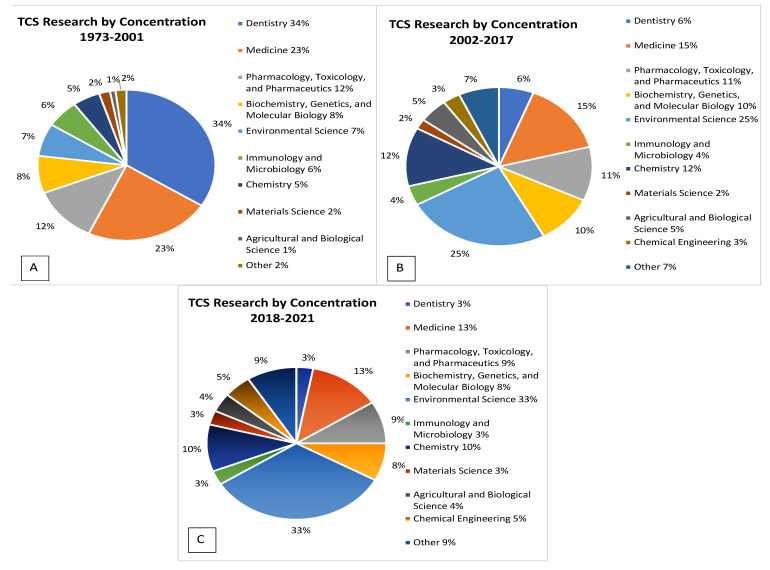
Concentrations of TCS-related research over three phases. Phase 1: 1973–2001. Phase 2: 2002–2017. Phase 3: 2018–2021. Values outside of the chart indicate the number of publications in each concentration. Percentages of these values can be found in the legend.

Analyzing the subject and concentration of TCS-related research in three main phases can outline a more detailed research trend and make clear what areas may need more focus (Figure 3). These pie charts outline the most common areas of research over three different significant periods related to TCS research. Each of these phases is based on spikes in produced publications seen in Figure 1 and major regulatory changes. These phases encompass large-scale changes in the use and production of TCS as well as policy changes. The first, 1973–2001, includes a heavy focus on TCS use in medical and dental settings. During this phase, 34% of research during this time was directed toward the topic of dentistry, while another 23% was aimed at TCS use in medical settings. All other topics form a minority during this phase of research, and at this point, the potential dangers of TCS were not widely known, allowing TCS to become more and more popular in use (Figure 3A). Between the years 1977 and 1998, yearly TCS production increased from 0.5–1.0 million pounds to up to 10 million pounds [2]. The second phase of research, 2002–2017, saw a stark shift in research focus. Dentistry (5%), and Medicine (15%), decreased substantially from the previous phase (together forming 57% of all TCS research), while Environmental Science (25%) became the focus of the effects of TCS (Figure 3B). The actual number of publications related to Medicine and Dentistry increased from 137 to 639 between the first and second phases. This is low, however, when compared to categories such as Environmental Science, which increased from a mere 82 publications to 779 in the second phase. During this time, topics related to the effects of TCS on Immunology and Biology began to grow, most likely due to the newfound concerns about TCS in heavy use. TCS production also hit its heaviest peak of 14 million pounds produced per year [2]. This sharp increase in production likely sparked concerns over environmental and health effects, which explains the shift in research focus. Publications produced at this time were related to environmental concerns, including antibiotic resistance and negative health effects, and may have played an important role in the eventual FDA regulations on TCS use. The third phase of research, post-FDA regulations (2018–2021), saw an even sharper increase in focus related to environmental concerns (Figure 3C). Research in Environmental Sciences became more dominant (33% of total research) during this time. This is because while the actual number of publications in the Environmental Science concentration remain stable between phase 2 and 3, Medicine, Dentistry, and other human health-related publications decreased substantially. Concerns over antimicrobial usage are at an all-time high, which explains the continued focus on the environmental effects of TCS.

### 3.3. Citation Analysis

**Figure 4:**
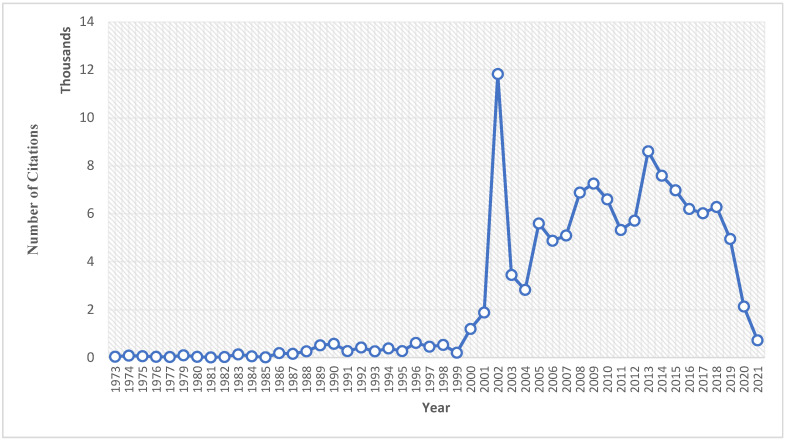
TCS-related citations per year.

A total of 3278 publications related to TCS and health led to a total of 123,534 citations (Figure 4). The trend of citations in addition to the publications indicated the recent explosion of research in the field. As early research related to TCS was limited, citations remained steady and low for a large portion of the early years (1973–2001). A significant increase in citations began after 2001, and there are a few notable outliers. In 2002, a sharp spike in citations might have been seen due to a few prominent articles published that year (Table 1). This sharp increase in citations was correlated with the increased presence of TCS in many products and the realization of its potential threat to environmental impact. Out of the topmost cited articles, 10 are related to wastewater treatment, the introduction of pharmaceuticals into the environment, and the effects of pollutants, such as TCS. However, none of these top-cited publications are geared towards the direct effects of TCS on human health, further displaying the lack of significant research in this area. This was noted by the FDA when making their decision in regulating the use of TCS in household items. The sources cited by the FDA while considering these issues, heavily favored topics related to antibiotic resistance and the overall efficacy of agents, such as TCS. Only a handful of those sources had any correlation with possible long-term effects, or effects of the bioaccumulation of TCS on human health. Even further, after cross-referencing the FDA’s published 2016 report, we found that only a few of the most cited articles in Table 1, as well as most published authors listed in Table 2, were cited as references in part of their decision to allow for the continued use of TCS in products other than hand soaps. Instead, the FDA acknowledged gaps in understanding the safety of TCS in issues such as dermal carcinogenicity, endocrine dysfunction, and even antibiotic resistance, noting that no additional research had been produced at that time that could definitively prove whether the agent could cause harm or not.

### 3.4. Journal Analysis

**Figure 5:**
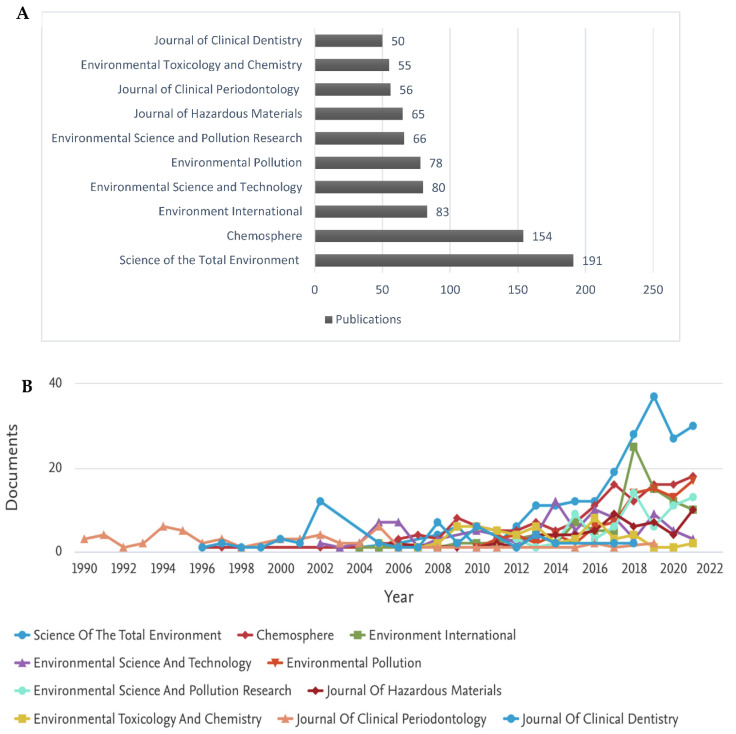
Most influential journals in TCS-related research. (**A**) Top 10 journals by the number of TCS-related publications for the years 1973–2021. (**B**) CiteScore™ analysis of top 10 Journals by publications for years 1990–2020.

The analysis of citations further demonstrates the research trends previously noted in other sections. The most influential journals related to the number of publications and citations are all directed toward environmental science. *Science of the Total Environment* and *Chemosphere* lead in publication number by almost two-fold in comparison to other journals of this type (Figure 5A). CiteScore™ is a feature of the Scopus database that can determine the impact of journals over time, based on influential publications produced and their respective citations [37]. The leading journals according to CiteScore^TM^ are *Environmental Science and Technology* and *Environment International* (Figure 5B). These journals led in impact factor over the last decade. *Environmental Science and Technology* was responsible only for the publication of 80 articles yet amounted to a mammoth 14,474 citations. The publications produced by the above journals are geared towards understanding the effects of TCS on the environment, including WWTP. Aside from the journals related to dentistry, no other journals were involved in topics such as medicine or the possible detrimental effects of TCS on human health.

### 3.5. Author and Global Network Analysis

The top 10 most prominent authors, by the number of publications, in the field of TCS research are shown (Table 2). As seen above, Antonia Calafat has published 65 articles and has a reported H-Index of 115. Calafat dedicates her work almost exclusively to Phthalates and other compounds, such as TCS, in consumer and personal care products [38]. She is currently affiliated with the National Center for Environmental Health and the CDC. William DeVizio, with a total of 19 publications involving TCS, is affiliated with the Colgate-Palmolive Company. DeVizio’s research in this field is focused on TCS’s role in toothpaste and its effectiveness against gingivitis and its safety for human use. His specific concentration of research began almost 10 years before the FDA approval of adding TCS into Colgate toothpaste products. Of the 10 authors in Table 2, 50% are affiliated with a Chinese institution, a fact that outlines China’s increased involvement in the field of TCS and its environmental and human health effects.

**Figure 6:**
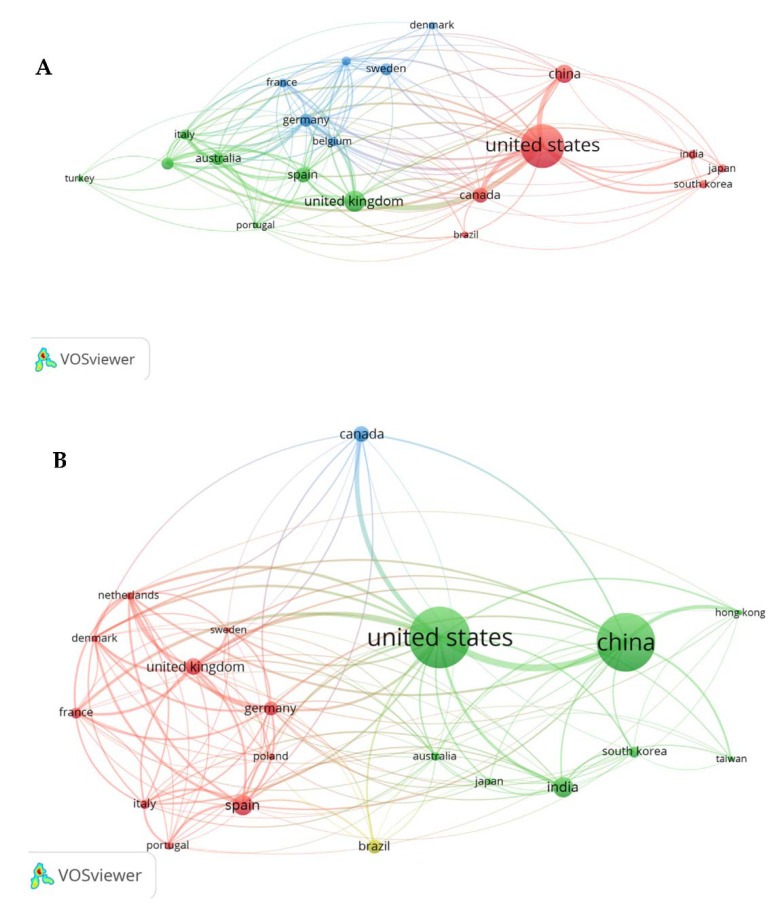
Network analysis of global TCS research from 2002–2017 (**A**) and 2018–2021 (**B**). Increased circle sizes are indicative of the increased number of citations. Colored lines represent co-citations between countries and the thickness of lines is indicative of the volume of co-citations thus the strength of collaborations.

This trend can be further visualized by network analysis (Figure 6), which displays a global network analysis trend over time. Note that the period 1973–2001 did not have enough collaborative research activities for meaningful network analysis outcomes. The above authors play a large role in this collaboration network and are responsible for China becoming a much more influential player in the field of TCS research over the last few years.

From the period of 2002–2017, the United States was the most influential country concerning TCS research by publications, citations, and overall impact. However, in recent years, from 2018–2021, China has grown to become a major powerhouse in the involvement of this type of research. Although they are still a distant second with the sheer volume of publications produced by the U.S. (Table 3), their extremely rapid increase demonstrates that they may grow to become more and more involved in fields that were recently of no concern. This seems to be the trend in most other scientific fields perhaps reflecting China’s increased economic capacity and investment in more basic sciences to meet its societal needs [39]. In addition, the number of publications produced does not always correspond with quality of research. Countries such as Australia and the United Kingdom may not have produced the most publications related to TCS overall, but in proportion to those articles’ citations, it is safe to assume that the research produced is effective.

### 3.6. Keyword Analysis

**Figure 7:**
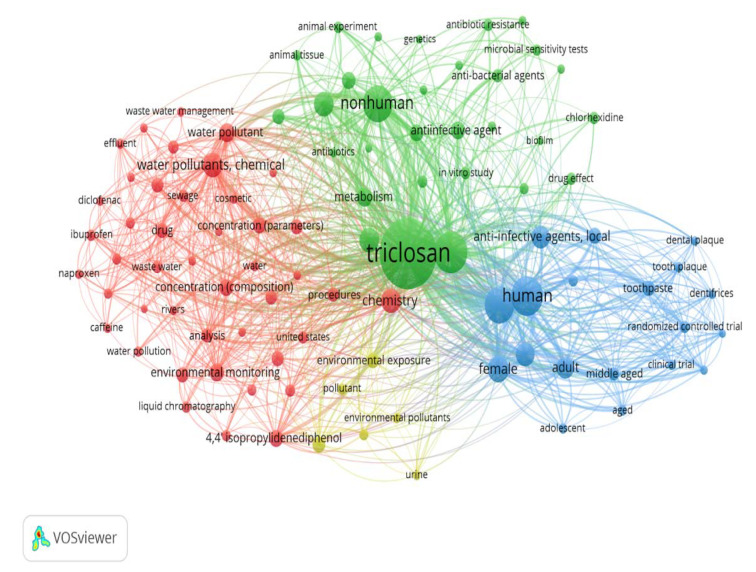
Network analysis by keywords used in TCS-related publications. Colors represent differing concentrations. Red represents “environmental impacts”. Green represents “antibiotic resistance”. Blue represents the use of “TCS in dentifrices”.

Keyword analysis networks are useful in displaying the main research focuses (Figure 7). Keywords were extracted from both titles and abstracts and compiled to create the most repetitive keywords used for each other. The most prominent keywords related to TCS research can be divided into three sections. Environmental impact keywords such as: “water pollutants”, “environmental monitoring”, “wastewater management” etc. The second section of keywords is related to TCS and antibiotic resistance, such as “anti-effective agent”, “antibacterial agent”, and “antibiotics”. The third main section of most prominent keywords relates to the use of TCS in dentifrices, including “tooth plaque”, “toothpaste”, and “clinical trials”. Very few keywords are related to the direct effects of TCS on human health, and the main concerns surrounding TCS usages, such as endocrine disruption, bioaccumulation, immune system suppression, or negative effects on prenatal development.

## 4. Conclusions

Triclosan (TCS) was eliminated from hand soaps in 2016 by the FDA but remains in a variety of everyday items, yet the effect on human health remain elusive. So, we have attempted to identify the research trends of TCS to determine whether research resources have been adequately spent to resolve the issue of uncertainty. TCS research has substantially increased over the last 50 years, but we determined that there are definitive gaps that remain in certain areas of focus. Although a large number of publications and citations have been produced, the vast majority are in relation only to the environmental impacts of TCS or its use in specific products, such as dentifrices and surgical devices. We found a significant lack of research in relation directly to TCS and human health in comparison with that related to its environmental impact. This, in conjunction with the FDA’s acknowledgment of a lack of definitive understanding of the role TCS could play in dermal carcinogenicity, endocrine dysfunction, and antibiotic resistance, proves the need for additional research in these fields. Since the current study is based on bibliometric analysis, it is focused on identifying overall trends, so we plan to provide a fuller picture of the collective understanding of TCS research in review paper format.

## Figures and Tables

**Table 1:** Most significant TCS-related research publications by the number of citations.

Publication	# of Citations	Journal	Year of Publication
Pharmaceuticals, hormones, and other organic wastewater contaminants in U.S. streams, 1999–2000: A national reconnaissance	6449	Environmental Science and Technology	2002
Recent Advances in Antimicrobial Treatments of Textiles	835	Textile Research Journal	2008
Guideline for hand hygiene in health-care settings: Recommendations of the healthcare infection control practices advisory committee and the HICPAC/SHEA/APIC/IDSA hand hygiene task force	678	Infection Control and Hospital Epidemiology	2002
Occurrence and fate of pharmaceutically active compounds in the environment, a case study: Höje River in Sweden	648	Journal of Hazardous Materials	2005
Microplastic moves pollutants and additives to worms, reducing functions linked to health and biodiversity	603	Current Biology	2013
Genome sequence and comparative analysis of the model rodent malaria parasite Plasmodium yoelii yoelii	601	Nature	2002
Pharmaceuticals and personal care products (PPCPs) in surface and treated waters of Louisiana, USA and Ontario, Canada	586	Science of the Total Environment	2003
Occurrence of some organic UV filters in wastewater, in surface waters, and in fish from Swiss lakes	552	Environmental Science and Technology	2005
epic2: National Evidence-Based Guidelines for Preventing Healthcare-Associated Infections in NHS Hospitals in England	532	Journal of Hospital Infection	2007
Pilot survey monitoring pharmaceuticals and related compounds in a sewage treatment plant located on the Mediterranean coast	467	Chemosphere	2007
Seasonal variations in concentrations of pharmaceuticals and personal care products in drinking water and reclaimed wastewater in Southern California	460	Environmental Science and Technology	2006
Co-occurrence of triclocarban and triclosan in U.S. water resources	452	Environmental Science and Technology	2005
Occurrence and environmental behavior of the bactericide triclosan and its methyl derivative in surface waters and in wastewater	436	Environmental Science and Technology	2002
Occurrence and reductions of pharmaceuticals and personal care products and estrogens by municipal wastewater treatment plants in Ontario, Canada	421	Science of the Total Environment	2006
Prenatal phenol and phthalate exposures and birth outcomes	420	Environmental Health Perspectives	2008
Structures of Novel Antimicrobial Agents for Textiles—A Review	413	Textile Research Journal	2010
Triclosan: Applications and safety	412	American Journal of Infection Control	1996
Analysis of Endocrine Disruptors, Pharmaceuticals, and Personal Care Products in Water Using Liquid Chromatography/Tandem Mass Spectrometry	396	Analytical Chemistry	2003
Triclosan offers protection against blood stages of malaria by inhibiting enoyl-ACP reductase of Plasmodium falciparum	395	Nature Medicine	2001
Urinary concentrations of triclosan in the U.S. population: 2003–2004	391	Environmental Health Perspectives	2008

**Table 2:** Most prominent authors by the number of TCS publications and H-index.

Author	# of Publications	H-Index	Affiliation
Calafat, A.M.	65	115	National Center for Environmental Health
Ying, G.G.	37	79	South China Normal University
Ye, X.	33	58	CDC, USA
Halden, R.U.	25	54	Arizona State University
Cai, Z.	24	67	Hong Kong Baptist University
Kannan, K.	21	123	NYU Grossman School of Medicine
Wang, X.	21	31	Wenzhou Medical University
Zhao, J.L.	20	50	South China Normal University
DeVizio, W.	19	31	Colgate-Palmolive Company
He, T.	19	20	Tongji Medical College

**Table 3:** Global research trends 1973–2021 by number of publications, number of citations, average publications per year, and average citations per publication.

Country	Total # Publications	Total # Citations	Avg. Publications/Year (1973–2022)	Avg. Citations Per Publication (1973–2022)
United States	1038	34,549	20.76	33.28
China	549	9415	10.98	17.15
United Kingdom	313	12,099	6.26	38.65
Spain	218	6577	4.36	10.64
Canada	179	5892	3.58	32.92
Germany	157	5259	3.14	33.50
India	153	2822	3.06	18.44
Brazil	127	1863	2.54	14.70
Australia	100	4991	2.00	49.91
France	98	2584	1.96	26.37

## Data Availability

Data are provided in the Supplementary Materials.

## Supplementary Materials

The following supporting information can be downloaded at: https://www.mdpi.com/article/10.3390/toxics10090523/s1, Table S1: Publications used in this study.
